# Azithromycin-Nonsusceptible *Shigella flexneri* 3a in Men Who Have Sex with Men, Taiwan, 2015–2016

**DOI:** 10.3201/eid2302.161260

**Published:** 2017-02

**Authors:** Ying-Shu Liao, Yen-Yi Liu, Yi-Chun Lo, Chien-Shun Chiou

**Affiliations:** Taiwan Centers for Disease Control, Taipei, Taiwan

**Keywords:** bacteria, shigella, dysentery, azithromycin, antimicrobial resistance, sexually transmitted infections, enteric infections, men who have sex with men, Taiwan, Shigella flexneri

## Abstract

We report an outbreak of azithromycin-nonsusceptible *Shigella flexneri* 3a infection in Taiwan associated with men who have sex with men. The bacterial strains belonged to the sublineage A of a recently reported outbreak lineage associated with men who have sex with men, characterized by reduced azithromycin susceptibility and circulation in shigellosis low-risk regions.

Shigellosis among men who have sex with men (MSM) is a major public health concern worldwide (*1**,**2*). *Shigella flexneri* serotype 3a was responsible for a prolonged MSM-associated outbreak in England and Wales initially detected in 2009 and ongoing at the time of study publication (*3*). More recently, *S. flexneri* 3a strains with a genetic lineage distinct from Africa- and Asia-associated lineages were identified among MSM in Europe, Australia, and North America (*4*). All strains in this lineage carried a mobile element with 4 antimicrobial resistance genes (*bla*_OXA-1_, *catA1*, *aadA1*, and *tetB*), and most recently emerging strains harbored a conjugative IncFII plasmid pKSR100, which conferred high-level resistance to azithromycin (*4*).

During 2005–2014, the incidence of domestically acquired shigellosis in Taiwan was low (0.7/100,000 persons) (*5*). Shigellosis is reported through the Web-based National Notifiable Disease Surveillance System (NDSS), which has been in operation since 1997 to monitor all notifiable diseases in Taiwan (*6**,**7*). The NDSS detected the first shigellosis outbreak in MSM during March–May 2015; the outbreak was determined to be caused by the global spread of a ciprofloxacin-resistant *S. sonnei* clone (*8*). During June 2015–May 2016, a total of 200 shigellosis cases were reported to the NDSS, of which 21 were domestically acquired *S. flexneri* 3a infections in northern and central Taiwan. All 21 of the *S. flexneri* 3a infections were in men 22–44 years of age, including 17 self-reported MSM. Of the 21 case-patients, most had reported other infections before *S. flexneri* 3a diagnosis: HIV (n = 16), 0–162 (median 49) months earlier; syphilis (n = 17), 0–85 (median 9.5) months earlier; gonorrhea (n = 6), 14–130 (median 26) months earlier; amebiasis (n = 2), 0–112 (median 56) months earlier; acute hepatitis A (n = 1), 0 months earlier; and *S. sonnei* infection (n = 1), 3 months earlier.

We tested the 21 *S. flexneri* 3a isolates for antimicrobial resistance by using a custom-made 96-well Sensititer MIC panel (Trek Diagnostic Systems Ltd., West Grinstead, UK) and the Etest kit (bioMérieux, Marcy l’Etoile, France). All 21 were resistant to ampicillin, chloramphenicol, streptomycin, and tetracycline, and 19 were nonsusceptible to azithromycin (MIC 64–96 μg/mL). 

We also used the PulseNet pulsed-field gel electrophoresis (PFGE) protocol to analyze the isolates (*9*) and compared the PFGE patterns with those of 30 *S. flexneri* 3a isolates isolated in Taiwan during 2000–2011. The NotI digested PFGE patterns of 20/21 of the isolates were identical to the earlier isolates, and the remaining isolate showed a highly similar pattern. Clustering analysis of the PFGE patterns by using BioNumerics version 6.6 software (Applied Maths, Sint-Martens-Latem, Belgium) revealed that the 21 isolates were genetically distant from the 2000–2011 *S. flexneri* 3a isolates. 

We used the Illumina MiSeq platform (Illumina, San Diego, CA, USA) for whole-genome sequencing of 4 isolates, of which 3 were azithromycin-nonsusceptible (codes R15.1162, R16.001, and R16.0013) and 1 was azithromycin-susceptible (R15.3406). We deposited the raw sequence reads in the National Center for Biotechnology Information Short Read Archive database (accession no. SRP080176; BioProject PRJNA335684). We used CLC Genomics Workbench version 9.0.1 (CLC bio, Aarhus, Denmark) for de novo assembly of the reads. We uploaded contigs for each isolate to the website of Center for Genome Epidemiology (http://www.genomicepidemiology.org/) and used its tools to identify sequence type (Multi Locus Sequnce Typing toolkit), plasmid type (PlasmidFinder), and antimicrobial resistance genes (ResFinder 2.1).

The 4 isolates were sequence type 245 (ST245), harbored plasmids belonging to incompatibility groups IncFII and Col, and had resistance genes *bla*_OXA-1_, *catA1*, *aadA1*, *tetB*, *ermB*, *mphA*, and *bla*_TEM_. Although isolate R15.3406 carried an intact *mphA* gene, it was phenotypically azithromycin susceptible. We compared genome single-nucleotide polymorphisms and constructed trees by using the CSI Phylogeny pipeline (https://cge.cbs.dtu.dk/services/CSIPhylogeny/) (*10*) to infer phylogenetic relationships between the 4 isolates and the 331 strains used in the study by Baker et al. (*4*). These analyses showed that the 4 isolates recovered in Taiwan belonged to the sublineage A of the MSM-associated outbreak lineage reported by Baker et al. (*4*).

In conclusion, we report an azithromycin-nonsusceptible *S. flexneri* 3a outbreak associated with MSM in Taiwan. The 21 isolates were genetically distant from the *S. flexneri* 3a isolates recovered in Taiwan from 2000–2011 and belonged to the sublineage A of the recently reported MSM-associated outbreak lineage characterized by reduced azithromycin susceptibility and circulation in shigellosis low-risk regions (*4*). The introduction of this MSM-associated *S. flexneri* 3a lineage into Taiwan in 2015 illustrates that the pathogen can spread rapidly across continents, possibly through intensified sexual networks among MSM (*2**,**8*). We recommend continued surveillance for antimicrobial resistance genes in *S. flexneri* to inform clinical management of shigellosis among MSM and public health interventions where needed, including appropriate antimicrobial drug stewardship.
